# The Immunomodulatory Role of Adjuvants in Vaccines Formulated with the Recombinant Antigens *Ov*-103 and *Ov*-RAL-2 against *Onchocerca volvulus* in Mice

**DOI:** 10.1371/journal.pntd.0004797

**Published:** 2016-07-07

**Authors:** Jessica A. Hess, Bin Zhan, April R. Torigian, John B. Patton, Nikolai Petrovsky, Tingting Zhan, Maria Elena Bottazzi, Peter J. Hotez, Thomas R. Klei, Sara Lustigman, David Abraham

**Affiliations:** 1 Department of Microbiology and Immunology, Sidney Kimmel Medical College, Thomas Jefferson University, Philadelphia, Pennsylvania, United States of America; 2 Department of Pediatrics, National School of Tropical Medicine, Baylor College of Medicine, Houston, Texas, United States of America; 3 Sabin Vaccine Institute and Texas Children’s Hospital Center for Vaccine Development, Houston, Texas, United States of America; 4 Department of Diabetes and Endocrinology, Flinders University, Adelaide, Australia; 5 Vaxine Pty Ltd, Flinders Medical Centre, Bedford Park, Adelaide, Australia; 6 Division of Biostatistics, Department of Pharmacology and Experimental Therapeutics, Thomas Jefferson University, Philadelphia, Pennsylvania, United States of America; 7 Department of Pathobiological Sciences, LSU School of Veterinary Medicine, Louisiana State University, Baton Rouge, Louisiana, United States of America; 8 Laboratory of Molecular Parasitology, Lindsley F. Kimball Research Institute, New York Blood Center, New York, New York, United States of America; University of Edinburgh, UNITED KINGDOM

## Abstract

**Background:**

In some regions in Africa, elimination of onchocerciasis may be possible with mass drug administration, although there is concern based on several factors that onchocerciasis cannot be eliminated solely through this approach. A vaccine against *Onchocerca volvulus* would provide a critical tool for the ultimate elimination of this infection. Previous studies have demonstrated that immunization of mice with *Ov-*103 and *Ov-*RAL-2, when formulated with alum, induced protective immunity. It was hypothesized that the levels of protective immunity induced with the two recombinant antigens formulated with alum would be improved by formulation with other adjuvants known to enhance different types of antigen-specific immune responses.

**Methodology/ Principal Findings:**

Immunizing mice with *Ov-*103 and *Ov-*RAL-2 in conjunction with alum, Advax 2 and MF59 induced significant levels of larval killing and host protection. The immune response was biased towards Th2 with all three of the adjuvants, with IgG1 the dominant antibody. Improved larval killing and host protection was observed in mice immunized with co-administered *Ov-*103 and *Ov-*RAL-2 in conjunction with each of the three adjuvants as compared to single immunizations. Antigen–specific antibody titers were significantly increased in mice immunized concurrently with the two antigens. Based on chemokine levels, it appears that neutrophils and eosinophils participate in the protective immune response induced by *Ov-*103, and macrophages and neutrophils participate in immunity induced by *Ov-*RAL-2.

**Conclusions/Significance:**

The mechanism of protective immunity induced by *Ov-*103 and *Ov-*RAL-2, with the adjuvants alum, Advax 2 and MF59, appears to be multifactorial with roles for cytokines, chemokines, antibody and specific effector cells. The vaccines developed in this study have the potential of reducing the morbidity associated with onchocerciasis in humans.

## Author Summary

In some regions in Africa, elimination of onchocerciasis may be possible with mass drug administration, although there is concern based on several factors that onchocerciasis cannot be eliminated solely through this approach. A vaccine against *Onchocerca volvulus* would provide a critical tool for the ultimate elimination of this infection. Previous studies have demonstrated that immunization of mice with two antigens induced protective immunity and it was hypothesized in the present study that the levels of protective immunity would be improved by formulation with other agents known to enhance immune responses. Protective immunity was observed in mice immunized with the two antigens using three different adjuvants. The vaccines developed in this study have the potential of reducing the morbidity associated with onchocerciasis in humans.

## Introduction

Onchocerciasis, caused by the filarial worm *Onchocerca volvulus*, is a neglected tropical disease (NTD) endemic predominantly in Africa. The Global Burden of Disease Study 2013 estimate indicates that 17 million people are currently infected with *O*. *volvulus* [1]. The disease, also referred to as river blindness, is an important cause of blindness, skin disease and chronic disability. Moreover, in children from Uganda and South Sudan, there are links between *O*. *volvulus* infection and a serious neurological disorder known as “nodding syndrome” [2, 3]. In some endemic regions evidence suggests that elimination of onchocerciasis may be possible with mass drug administration (MDA) of ivermectin [4]. Several significant obstacles must be overcome before complete elimination in Africa can be achieved. First, it has been estimated that elimination will require 14–35 years of continuous treatment [5, 6]. Furthermore, based on animal and human studies, susceptibility to reinfection increases after treatment [7–9]. In addition, there have been several reports which suggest that *O*. *volvulus* in some regions in Africa may have developed resistance to ivermectin [10–18]. Finally, MDA of ivermectin is not possible in large areas of central Africa where loiasis is co-endemic, because of the risk of developing severe adverse reactions to the treatment including encephalopathy in individuals with high level of *Loa loa* microfilaremia [19]. Therefore, there is a growing consensus supported by mathematical modeling, that onchocerciasis in Africa will not be eliminated within the original proposed timeframes using MDA alone. It has been estimated now that elimination would require 1.15 billion treatments up until 2045, while other estimates suggest that onchocerciasis cannot be eliminated solely through MDA with ivermectin [20, 21]. A vaccine against onchocerciasis, to complement the present control measures, would therefore provide a critical tool for the ultimate elimination of this infection from humans [22, 23]. Mathematical modeling of the impact of vaccination against *O*. *volvulus* suggests that a prophylactic vaccine would reduce disease burden related to onchocerciasis in regions where ivermectin cannot be administered safely and would decrease the chance of re-emergence of the parasite after mass drug administration has been stopped [24].

A mouse model was developed for studying immunity to *O*. *volvulus* in which larvae are implanted subcutaneously in mice within diffusion chambers [25]. Protective immunity was demonstrated in this model following immunization of mice with irradiated third-stage infective larvae of *O*. *volvulus* [26–30]. The model was also used to identify recombinant antigens that could be used in a vaccine against infection with larval *O*. *volvulus* [31, 32]. When some of these recombinant antigens were produced under standardized conditions, two antigens emerged as lead vaccine candidates, *Ov-*103 and *Ov-*RAL-2, based on their ability to induce significant levels of protective immunity after immunization using alum as the adjuvant [33]. This observation was confirmed in gerbils immunized with the *Brugia malayi* proteins *BM-103* and *Bm*-RAL-2, which are orthologous to the *O*. *volvulus* proteins. Vaccination with *BM-103* and *Bm*-RAL-2, with alum as the adjuvant, induced protective immunity to infection with *B*. malayi in gerbils [34]. Both proteins are highly conserved within nematodes and homologs of these antigens have been shown to induce protective immunity to other nematodes [35–41]. The functional properties of Ov-103 and Ov-RAL-2 are currently unknown, however, both proteins are localized on the surface and glandular esophagus of third-stage larvae (L3) as well as in the hypodermis and cuticle of adult worms and on the surface of microfilariae [34, 42, 43].

The primary objective of the present study was to test the hypothesis that the levels of protective immunity induced with *Ov-*103 and *Ov-*RAL-2 formulated with alum could be increased by formulating these antigens with immune enhancing adjuvants. Five adjuvants (alum, Advax 1, Advax 2, CpG oligonucleotide (CpG), and MF59) were selected for comparative analysis based on their ability to induce different types of immune responses. Alum, the most commonly used adjuvant in human vaccines, elicits strong humoral immune responses, which are mediated primarily by IgG1 [44, 45]. This adjuvant stimulates strong Th2 responses but does not induce cell-mediated responses [46–50]. Injection of alum into mice increased the expression of the neutrophil-specific chemokines CXCL1(KC) and CXCL2, the monocyte-specific chemokines CCL2 (MCP-1) and CCL4 (MIP-1β) and the eosinophil chemokines CCL11 (eotaxin-1) and CCL24 (eotaxin-2) [51, 52]. Alum appears to act mainly on macrophages and monocytes to induce secretion of chemokines involved with cell recruitment from the blood into peripheral tissues [53].

Advax 1 is a novel polysaccharide adjuvant derived from delta inulin [54] that is under development for use in humans [55, 56]. It is successful at inducing a mixed Th1/Th2 associated IgG1 and IgG2a antibody response [57], as well as Th1, Th2 and Th17 cytokine responses [58, 59]. Advax 2 is comprised of delta inulin formulated with a small amount of CpG, a TLR9 agonist which shifts some of the responses to Th1 while retaining the Th2 response. It also potently induces CD8^+^ CTL and generally also gives the highest overall IgG response due to induction of a broad combination of IgG1, IgG2 and IgG3 antibodies. CpG is a strongly Th-1 biased adjuvant that typically gives an IgG response comprised predominantly of IgG2 antibodies [60, 61].

MF59, an oil-in-water emulsion adjuvant, has been established as safe and potent adjuvant for use in human vaccines [62, 63]. This adjuvant induces a mixed Th1/Th2 response in humans and animals [64, 65] with both antigen-specific IgG1 and IgG2a antibodies produced [66], and has been shown to be more potent than alum for the induction of both antibody and CD4^+^ responses [67, 68]. MF59 appears to act on macrophages, monocytes and granulocytes to induce secretion of MCP-1, CCL3 (MIP-1α), MIP-1β, all involved with cell recruitment from blood into peripheral tissue [53].

Two criteria were used for measuring protective immunity in the present studies. The first criterion was killing of parasites as represented by the comparison between the mean numbers of larvae surviving in control vs. immunized mice. This metric assesses the ability of the induced effector responses to kill worms and the capacity of all or part of the worm population to evade the killing response. Parasite reduction is of particular importance in the case of onchocerciasis since reducing worm burden would have a beneficial effect on health status, without a requirement for achieving sterile immunity. The second measure was host protection, where the objective was to determine the number of immunized mice that had parasite recovery below the 95% confidence interval seen in the control mice. This metric describes the efficacy of the vaccine, by estimating what percent of vaccinees benefited from the prophylactic vaccine. Reduced levels of infection within a population will likely enhance control of new infections and thus disease within the endemic region.

A reductionist experimental approach was used in this study, with the initial screening of all five adjuvants performed using *Ov-*103 as the antigen. Adjuvants that were successful at inducing immunity with *Ov-*103 were then tested with *Ov-*RAL-2 and finally in a vaccine consisting of co-administered *Ov-*103 and *Ov-*RAL-2. Analyses were performed to identify immune correlates and potential mechanisms of protective immunity induced by the antigens, individually or when co-administered, with the selected adjuvants. Once again, a reductionist experimental approach was undertaken; all classes and sub-classes of antibody responses were initially tested in mice immunized with *Ov-*103. Analyses of antibody response to *Ov-*RAL-2 and the *Ov-*103/*Ov-*RAL-2 co-administered vaccine were then limited to the antibody subclasses that yielded positive responses to *Ov-*103. Likewise, cytokine and chemokine responses were measured in mice immunized with *Ov-*103 and the positive sub-sets measured in subsequent experiments. Three adjuvants were identified that induced protective immunity with *Ov-*103 and *Ov-*RAL-2 and with the co-administered vaccine. Immunological correlates of protective immunity were also observed based on unique antibody, cytokine and chemokine signatures.

## Materials and Methods

### Source of parasites and mice

*O*. *volvulus* L3 were collected from black flies (*Simulium damnosum*) that were fed on consenting infected donors (Protocol 320 was approved by the New York Blood Center and the Medical Research Station, Kumba, Cameroon IRBs). After seven days the flies were dissected to collect the developed L3, which were cleaned and cryopreserved as previously described [69].

Male BALB/cByJ mice, 6–8 weeks of age, were purchased from The Jackson Laboratory (Bar Harbor Maine). All mice were housed in micro-isolator boxes in rooms that were pathogen free and under temperature, humidity and light cycle controlled conditions in the Laboratory Animal Sciences Facility at Thomas Jefferson University. Mice were fed autoclavable rodent chow and given water ad libitum.

### Animal ethics

All experimental procedures were performed in compliance with the ethical and regulatory standards set by the NIH for animal experimentation. The animal use protocol (00136) was approved by the Thomas Jefferson University Institutional Animal Care and Use Committee. The animal care and use protocol adhered to the “Guide for the Care and Use of Laboratory Animals” published by the National Research Council, USA.

### Production of antigens

Based on previous studies, *Ov-*103 was expressed in PichiaPink yeast and *Ov-*RAL-2 in *Escherichia coli*. Antigens were prepared and analyzed as previously described [33]. In addition, Ov-RAL-2 with His-tag at C-terminus was expressed in *E*. *coli* BL21, purified with nickel column and endotoxin removed with a Q anion exchange column. The level of endotoxin in the final products was less than 20EU/mg (13.2–19.3 EU/mg).

### Immunization and challenge protocol

For experiments testing individual antigens, mice were immunized with 25 μg of the produced vaccine antigen formulated with each of the five different adjuvants in a 100 μl total volume preparation as per the manufacturer's directions. The alum immunization consisted of 50% v/v of vaccine antigen in TBS and 1:5 Rehydragel LV (alum) in TBS (General Chemical, Parsippany, NJ). Advax 1, Advax 2, and CpG (Vaxine Pty Ltd, Adelaide, South Australia) were used at 1 mg of Advax 1 or Advax 2 or 10 μg of CpG mixed with vaccine antigen in TBS immediately prior to injection. For vaccines formulated with MF59 (Novartis Vaccines, Cambridge, MA), 50 μl vaccine antigen in TBS was mixed 1:1 v/v with the adjuvant. Mice were immunized intramuscularly with 50 μl of the formulated vaccines in each caudal thigh. The *Ov-*103/*Ov-*RAL-2 co-administered vaccine consisted of 25 μg of each vaccine antigen, formulated with adjuvant for a total of 50 μl; *Ov-*103 was injected in the left caudle muscle and *Ov-*RAL-2 in the right caudal muscle. Immunization was followed by two booster injections 14 and 28 days later.

Cryopreserved L3 were defrosted in a two-step process, 15 minutes on dry ice followed immediately by a 37° water bath. The thawed L3 were then washed 5 times in a 1:1 mixture of NCTC-135 and Iscove's modified Dulbecco's medium supplemented with 100 U penicillin, 100 μg streptomycin, 100 μg gentamicin and 30 μg of chloramphenicol per ml. Diffusion chambers were constructed from 14 mm Lucite rings covered with 5.0 μM pore-size Durapore membranes (EMDMiIIipore, Billerca, CA) and fused together using an adhesive containing a 1:1 mixture of 1,2-dichloroethane (Fisher Scientific, Pittsburg, PA) and acryloid resin (Rohm and Haas, Philadelphia, PA). The constructed diffusion chambers were then sterilized via 100% ethylene oxide followed by 12 hr aeration.

Fourteen days after the final booster, mice were challenged using a diffusion chamber containing 25 L3. The diffusion chambers were implanted in a subcutaneous pocket on the rear flank of the mice. The diffusion chambers were recovered 21 days later and larval survival was calculated based on the mobility and morphology of the remaining larvae. Protective immunity was evaluated by two different methods: (1) Percent reduction of larvae, calculated by: ((Average worm survival in control mice—Average worm survival in immunized mice) ÷ Average worm survival in control mice) x 100. (2) Host protection, calculated by: (Number of immunized mice with parasite recovery levels below the 95 confidence interval of parasite recovery in control mice ÷ total number of immunized mice) x 100. Cells within the diffusion chamber were collected, placed onto slides by centrifugation using a Cytospin 3 (Shandon Inc, Pittsburgh, PA), and then stained and analyzed for differential cell counts using Hemastain 3 (Fisher Scientific).

### ELISA

Serum for antigen-specific antibody analyses was collected when mice received challenge infections within diffusion chambers and at the conclusion of the experiment. lgG1, lgG2a, lgG2b, lgG3, IgM and IgE were measured in mice immunized with *Ov-*103 with each of the five adjuvants. Antigen-specific lgG1, lgG2a and lgG2b responses were measured in mice immunized with *Ov-*RAL-2 and co-administered *Ov-*103/*Ov-*RAL-2 formulated alone or with either alum, Advax 2 or MF59. Maxisorp 96-well plates (Nunc Nalgene International, Rochester, NY) were coated with 2 μg /ml of *Ov-*103 or *Ov-*RAL-2 in 50 mM Tris-CI coating buffer pH 8.8 overnight 4°C. Plates were washed with deionized water between each step. Borate buffer solution (BBS) (0.17 M boric acid, 0.12 M NaCl, 0.5% Tween-20, 0.025% bovine serum albumin, I mM EDTA, pH 8.2) was used to block the plates for 30 min at room temperature. Individual sera were diluted to an appropriate starting concentration with BBS and serially diluted; plates were sealed and incubated at 4°C overnight. Biotinylated anti-lgG1, -lgG2a, -IgG2b, -IgG3 and -IgE (BD Biosciences, San Jose, CA) and -IgM (Vector Labs, Burlingame, CA) antibodies were diluted 1:250 in BBS and incubated for 1 hr at room temperature. ExtrAvidin PX (Sigma, St. Louis, MO) was diluted 1:1000 in BBS and added for 30 min at room temperature. After the final wash, one component ABTS peroxidase substrate (KPL, Gaithersburg, MD) was added and optical densities were read after 30 min at 405 nm in an iMark Microplate reader (Bio-Rad, Hercules, CA). Endpoint titers were calculated as the lowest serum dilution from experimental animals that had an optical density reading three times higher than the lowest optical density recorded for control serum.

### Spleen cell stimulation

One week after the diffusion chamber recovery, spleens from control and immunized mice were aseptically removed and made into single cell suspensions. Cells were cultured in a 96-well plate at a concentration of 2x10^6^/well. The cells were stimulated with either 10 μg of *Ov-*103, *Ov-*RAL-2, media control or with anti-CD3 mAb (BD Biosciences) which was pre-coated at 0.5 μg/ml for 2h at 37°C. Each well also received 0.5 μl of anti-IL-4r (BD Biosciences) [70]. Cells were incubated at 37°C for 3 days, after which supernatants were collected and frozen at -20°C.

#### Cytokine and chemokine measurements by Luminex

Supernatants from stimulated spleen cell and the fluid from diffusion chambers recovered from mice immunized with *Ov-*103 formulated with the five adjuvants were analyzed using Milliplex Map Kit magnetic bead panels as per the manufacturer's protocol (EMDMiIIipore). Plates were analyzed on a MAGPIX Luminex machine (Austin, TX). All analyte concentrations were calculated using Milliplex Analyst software (EMDMiIIipore). In the initial studies, 22 cytokines and 6 chemokines were measured in both the supernatants from stimulated spleen cell and the fluid from diffusion chambers. Elevated responses were recorded for 9 cytokines in the spleen cell supernatants and 5 chemokines in diffusion chamber fluid, as compared to controls. Analysis of cytokine and chemokine levels in subsequent studies, with mice immunized with *Ov-*RAL-2 alone or in the co-administered vaccine, were limited to the 9 cytokines in the spleen cell supernatants and 5 chemokines in diffusion chamber fluid (Table 1, S1 and S2 Tables).

**Table 1:** Cytokines and chemokines measured by Luminex.

Cytokines			Chemokines	
Spleen Cells			Diffusion Chamber	
Elevated	Equal	Not Detected	Elevated	Not Detected
IFN-ƴ	IL-1α	IL-12p40	Eotaxin	Rantes
IL-2	IL-3	IL-12p70	KC	
IL-4	IL-22	IL-17/IL-25	MCP-1	
IL-5	IL-28β	IL-21	MIP-1α	
IL-6	TNF-α	IL-23	MIP-1β	
IL-10	GM-CSF	IL-33		
IL-13		TNF-β		
IL-17A				
IL-17F				

Cytokines and chemokines measured in spleen cell supernatants and diffusion chamber fluid by Milliplex Map Kit magnetic bead panels on MAGPIX Luminex. Results presented are for mice immunized with *Ov-*103, with cytokines only detected in the antigen-stimulated spleen cell supernatants and chemokines only detected in the diffusion chamber fluid. Elevated = increased levels in immunized mice as compared to controls. Equal = equal levels in immunized and control mice. Not detected = levels not detected from control and immunized mice. Subsequent studies with *Ov-*RAL-2 were limited to measurement of the 9 elevated cytokines from the spleen cell supernatants and 5 elevated chemokines from the diffusion chamber fluid.

### Statistical analysis

All experiments consisted of 5–6 mice per group with the experiments performed at least twice with consistent results between experiments. Data presented are cumulative from all experiments. Data were analyzed for parasite killing by multifactorial analysis of variance ANOVA in Systat v.ll (Systat Inc., Evanstown, IL). Probability values less than 0.05 were considered statistically significant. Bootstrap statistical analysis of host protection was performed using R package "boot". Bootstrap sample means were estimated from the control groups and the lower bound of the 95% confidence interval reported. A kernel density estimate of the vaccine group was calculated and the percentage below the bootstrap 95% confidence interval calculated.

## Results

### Role of adjuvants in the induction of protective immunity

**Fig 1:**
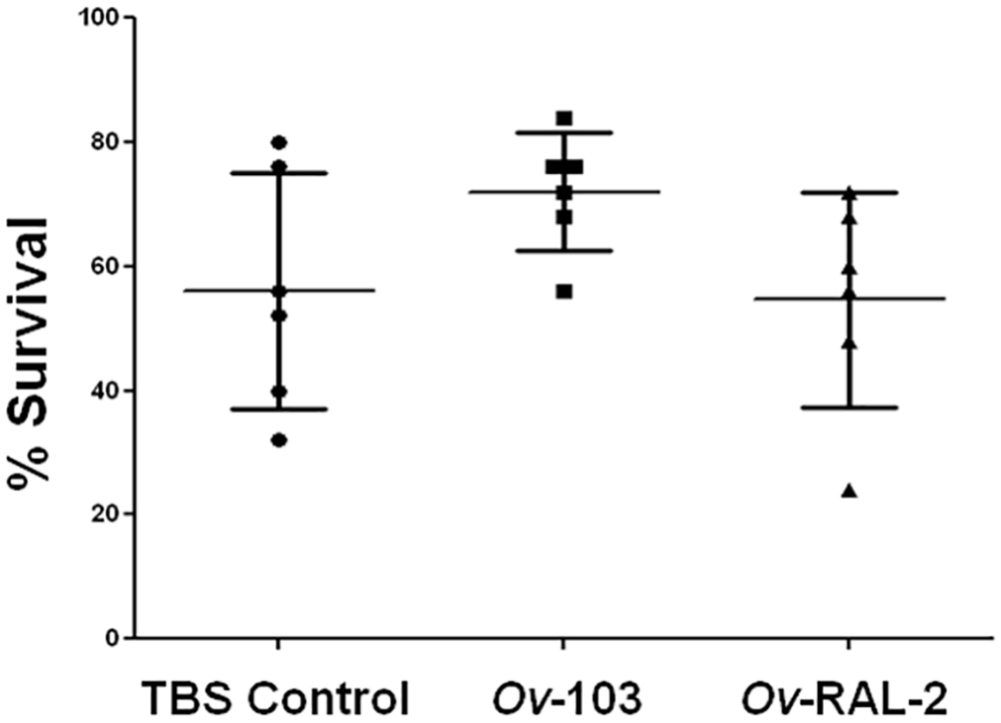
Survival of *Onchocerca volvulus* in mice immunized with *Ov-*103 or *Ov-*RAL-2 without adjuvant. Effect of immunization with *Ov-*103 or *Ov-*RAL-2 without adjuvant on the development of protective immunity to *Onchocerca volvulus* larvae in mice. Each dot represents percent larval recovery from an individual animal. Data presented are mean ± standard error.

BALB/cByJ mice were immunized intramuscularly three times with *Ov-*103 or *Ov-*RAL-2 without adjuvant. Control and immunized mice received challenge infections within diffusion chambers, and there was no evidence of protective immunity in the immunized mice (Fig 1). Cell migration into the diffusion chambers was equivalent between control and immunized groups with 5 x 10^5^ ± 8 x 10^5^ cells found within the parasite microenvironment. The differential distribution of cells found within the diffusion chamber was neutrophils (48 ± 18%), macrophages (48 ± 19%), lymphocytes (0 ± 0%) and eosinophils (5 ± 5%) in all groups of mice regardless of treatment status.

**Fig 2:**
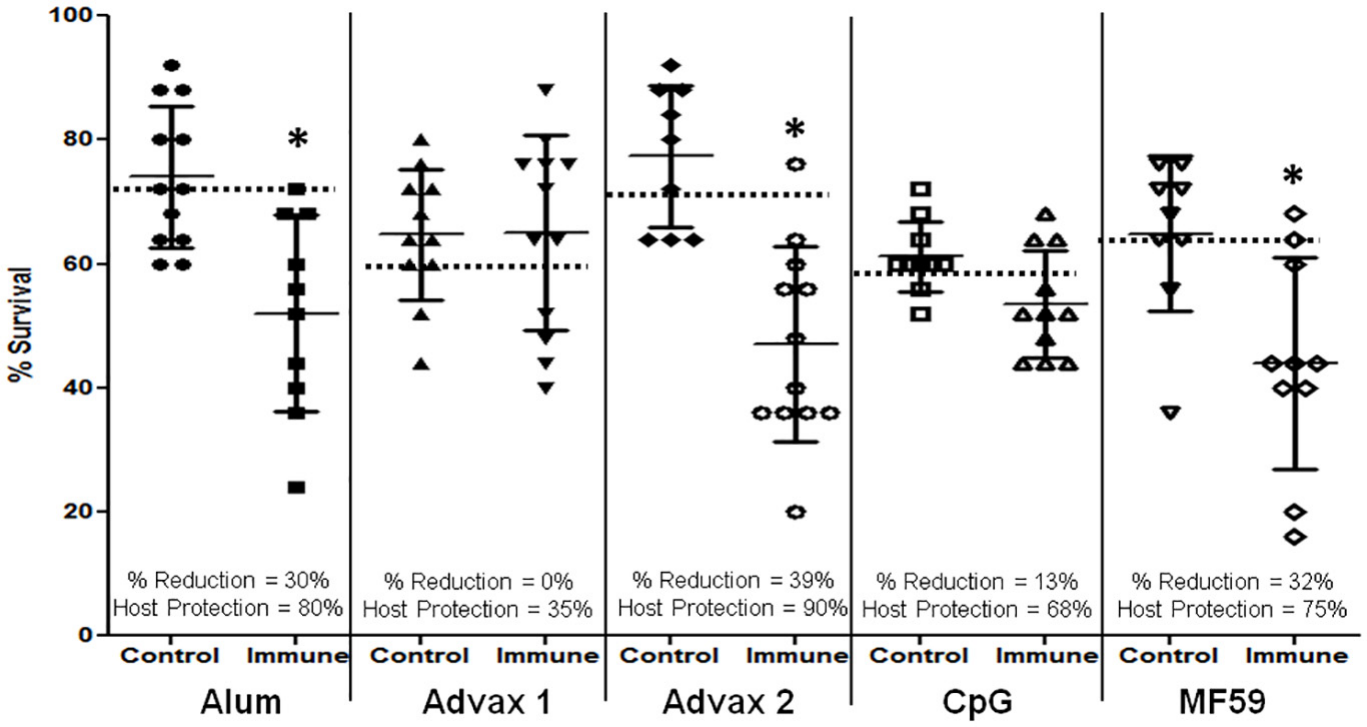
Survival of *Onchocerca volvulus* in mice immunized with *Ov-*103 with five different adjuvants. Effect of immunization with *Ov-*103 formulated with the adjuvants alum, Advax 1, Advax 2, CpG or MF59 on the development of protective immunity to *Onchocerca volvulus* larvae in mice. Each dot represents percent larval recovery from an individual animal. Data lines presented are mean ± standard error. Asterisk represents statistical difference in larval recoveries, p value ≤ 0.05. Dotted line is placed at the 95^th^ confidence interval for parasite recovery from control animals. % Reduction = percent reduction in parasite survival in immunized mice as compared to controls. Host Protection = percentage of mice in the immunized group with parasite recovery levels below the 95^th^confidence interval for parasite recovery from control animals.

Mice were immunized with *Ov-*103 formulated with one of the following five adjuvants: alum, Advax 1, Advax 2, CpG or MF59. Immunization with *Ov-*103 in combination with alum, Advax 2 and MF59 induced statistically significant reductions of larval survival (Fig 2). Mice immunized with *Ov-*103 formulated with alum had reductions in mean larval survival of 30% and host protection levels of 80%, with Advax 2 they had a 39% reduction in larval survival and 90% host protection and with MF59 they had a 32% reduction in larval survival and 75% host protection. Vaccination of mice with *Ov-*103 formulated with Advax 1 or CpG as adjuvants did not result in significant reductions in parasite survival yet they were associated with 35% and 68% host protection, respectively (Fig 2). Cell recruitment into diffusion chambers in control and immunized mice were comparable between all adjuvants with 1.8 x 10^6^ ± 1.6 x 10^6^ total cells and differential distribution of cells of neutrophils (56 ± 17%), macrophages (39 ± 16%), lymphocytes (1 ± 1%) and eosinophils (4 ± 4%).

**Fig 3:**
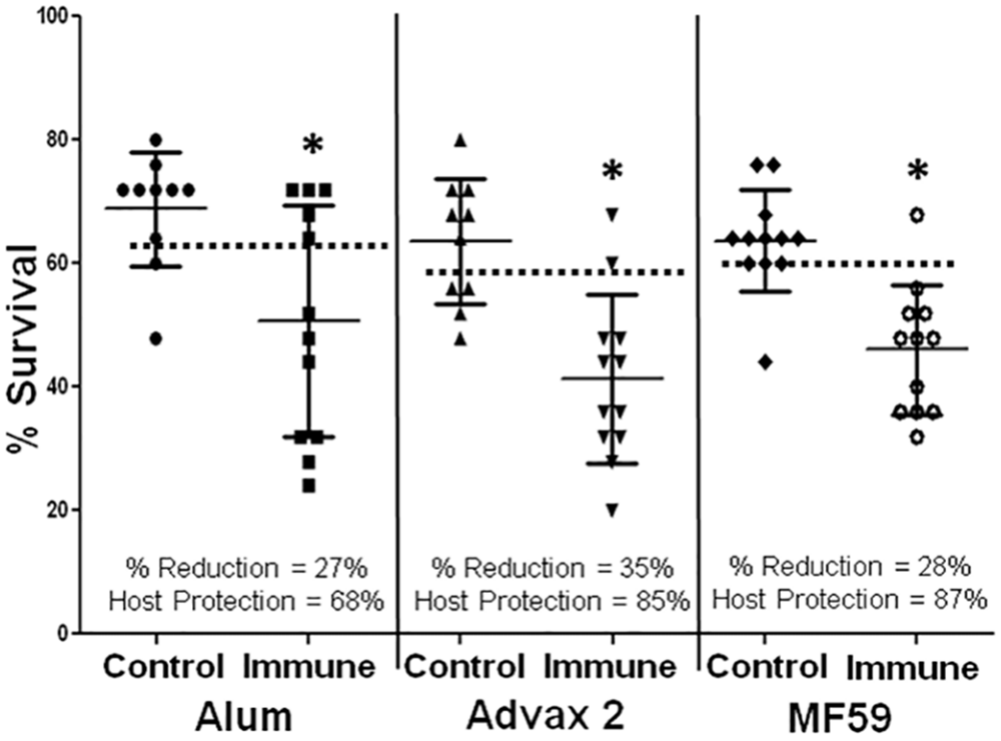
Survival of *Onchocerca volvulus* in mice immunized with *Ov-*RAL-2 with three different adjuvants. Effect of immunization with *Ov-*RAL-2 formulated with the adjuvants alum, Advax 2 or MF59 on the development of protective immunity to *Onchocerca volvulus* larvae in mice. Each dot represents percent larval recovery from an individual animal. Data lines presented are mean ± standard error. Asterisk represents statistical difference in larval recoveries, p value ≤ 0.05. Dotted line is placed at the 95^th^ confidence interval for parasite recovery from control animals. % Reduction = percent reduction in parasite survival in immunized mice as compared to controls. Host Protection = percentage of mice in the immunized group with parasite recovery levels below the 95^th^confidence interval for parasite recovery from control animals.

*Ov-*RAL-2 was tested as a vaccine in combination with the three adjuvants that induced protective immunity with *Ov-*103, specifically alum, Advax 2 and MF59. Immunization of mice with *Ov-*RAL-2 formulated with each of these three adjuvants induced statistically significant reductions in larval survival (Fig 3). Mice immunized with *Ov-*RAL-2 formulated with alum had reductions in mean larval survival of 27% and 68% host protection, with Advax 2 mice had a 35% reduction in larval survival and 85% host protection, and with MF59 mice had a 28% reduction in larval survival and 87% host protection (Fig 3). Cell recruitment into the diffusion chamber was comparable between all adjuvants with 1.4 x 10^6^ ± 1.7 x 10^6^ total cells and differential distribution of cells neutrophils (46 ± 17%), macrophages (49 ± 17%), lymphocytes (0 ± 1%) and eosinophils (5 ± 4%).

**Fig 4:**
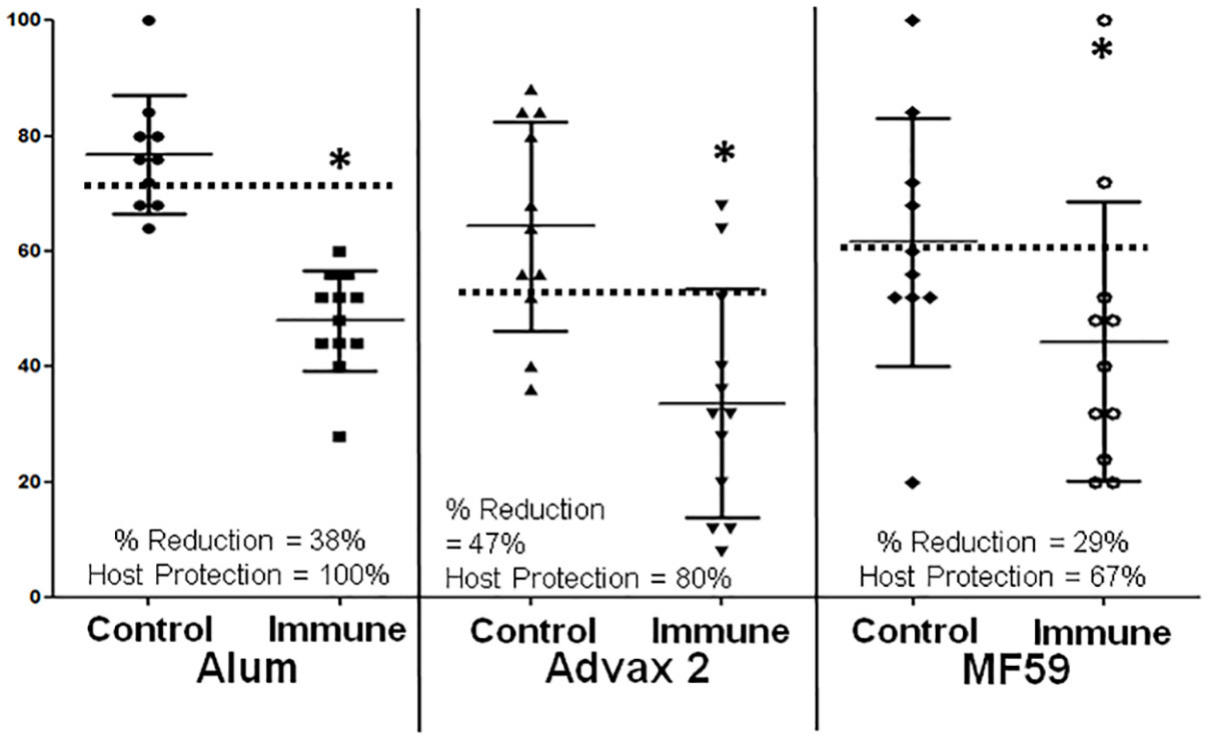
Survival of *Onchocerca volvulus* in mice immunized with *Ov-*103 and *Ov-*RAL-2 co-administered with three different adjuvants. Effect of immunization with the *Ov-*103 and *Ov-*RAL-2 co-administered vaccines formulated with the adjuvants alum, Advax 2 or MF59 on the development of protective immunity to *Onchocerca volvulus* larvae in mice. Each dot represents percent larval recovery from an individual animal. Data lines presented are mean ± standard error. Asterisk represents statistical difference in larval recoveries, p value ≤ 0.05. Dotted line is placed at the 95^th^ confidence interval for parasite recovery from control animals. % Reduction = percent reduction in parasite survival in immunized mice as compared to controls. Host Protection = percentage of mice in the immunized group with parasite recovery levels below the 95^th^confidence interval for parasite recovery from control animals.

Mice were immunized with co-administered *Ov-*103/*Ov-*RAL-2 formulated with alum, Advax 2 and MF59. Immunization of mice with *Ov-*103 and *Ov-*RAL-2 using all three adjuvants induced statistically significant reductions in larval survival (Fig 4). Mice immunized with the two antigen co-administered vaccine formulated with alum, had reductions in mean larval survival of 38% and 100% host protection, with Advax 2 mice had a 47% reduction in larval survival and 80% host protection and with MF59 mice had a 29% reduction in larval survival and 67% host protection (Fig 4). Cell recruitment into the diffusion chamber was comparable between all adjuvants with 1.0 x 10^6^ ± 1.1 x 10^6^ total cells and differential distribution of cells neutrophils (40 ± 14%), macrophages (56 ± 15%), lymphocytes (0 ± 0%) and eosinophils (4 ± 5%).

### Antibody responses in mice immunized with Ov-103 or Ov-RAL-2

Immunization of mice with *Ov-*103 or *Ov-*RAL-2 without adjuvant did not induce a significant IgG antibody response to either of the antigens. Antibody responses in mice immunized with *Ov-*103 formulated with each of the five adjuvants, were measured in serum recovered from mice at study termination. Mice immunized with *Ov-*103 formulated with each of the five adjuvants had positive IgG1 responses, with CpG inducing the lowest endpoint titer. *Ov-*103-specific IgG2a responses were only discernible in mice immunized with Advax 2 as the adjuvant (Table 2A). All immunized mice were negative for antigen-specific IgG2b, IgG3 and IgE. Control mice and mice immunized with *Ov-*103 formulated with any of the five adjuvants had equivalent antigen-specific IgM responses. Analysis of serum, recovered at the same time point, from mice immunized with *Ov-*RAL-2 was limited to IgG1, IgG2a and IgG2b. Mice immunized with *Ov-*RAL-2 formulated with the adjuvants alum, Advax 2 or MF59 developed positive IgG1, IgG2a and IgG2b antigen-specific responses. Enhanced IgG2a and IgG2b responses were observed in mice immunized with *Ov-*RAL-2 formulated with Advax-2 (Table 2B). When antigen-specific antibody responses were measured in mice immunized with the co-administered vaccines formulated with each of the three adjuvants, the *Ov-*103 and *Ov-*RAL-2 antigen-specific IgG1 responses developed were significantly elevated as compared to those seen in mice immunized with the corresponding single antigen vaccine. Antigen-specific IgG2a endpoint titers to *Ov-*103 and *Ov-*RAL-2 were only elevated in mice immunized with Advax 2 as the adjuvant in the co-administered vaccines, as compared to mice immunized with the single vaccines. Similarly, the IgG2b endpoint titers to *Ov-*RAL-2 were elevated in mice immunized with the co-administered vaccines with Advax 2 as the adjuvant, as compared to mice immunized with the *Ov-*RAL-2 formulated with Advax 2 (Table 2C).

**Table 2:** Antibody endpoint titers from mice immunized with *Ov-*103, *Ov-*RAL-2, or co-administration of *Ov-*103 and *Ov-*RAL-2 with adjuvant.

**A.**	**Ov-103**		
	**IgG1**	**IgG2a**	**IgG2b**
**Alum**	5,246 ± 4,538	ND	ND
**Advax 1**	3,772 ± 2,602	ND	ND
**Advax 2**	6,671 ± 3,893	773 ± 675	ND
**Advax 3**	285 ± 87	ND	ND
**MF59**	7,254 ± 5,518	ND	
**B.**	**Ov-RAL-2**		
	**IgG1**	**IgG2a**	**IgG2b**
**Alum**	33,250 ± 23,065	390 ± 504	88 ± 11
**Advax 2**	33,322 ± 44,332	11,599 ± 7,991	750 ± 519
**MF59**	6,635 ± 3,743	390 ± 504	88 ± 11
**C.**	**Ov-103**		
	**IgG1**	**IgG2a**	**IgG2b**
**Alum**	52,929 ± 49.748	ND	ND
**Advax 2**	29,679 ± 29,034	7,986 ± 12,117	267 ± 289
**MF59**	32,042 ± 36,608	ND	260 ± 308
	**Ov-RAL-2**		
	**IgG1**	**IgG2a**	**IgG2b**
**Alum**	155,971 ± 82,299	354 ± 396	82 ± 92
**Advax 2**	84,890 ± 70,963	22,177 ± 25,587	4,289 ± 6,492
**MF59**	41,962 ± 27,352	180 ± 112	100 ± 63

IgG1, IgG2a and IgG2b antibody endpoint titers from mice immunized with *Ov-*103 (A), *Ov-*RAL-2 (B) or co-administration of *Ov-*103 and *Ov-*RAL-2 (C) with adjuvant. Serum samples were taken at the conclusion of the experiment. Values presented are mean ± standard deviation.

Pre-challenge serum was collected from all mice and antibody class and sub-class responses were measured. It was determined that the type and magnitude of the responses mirrored those measured at study termination. Correlation analyses comparing parasite recovery numbers and antibody endpoint titers for both pre- and post-challenge serum did not reveal consistent significant levels of statistical correlation.

### Cytokine and chemokine responses in mice immunized with Ov-103 or Ov-RAL-2

Twenty eight analytes were measured in both *ex-vivo* spleen cell stimulation supernatants and fluid from diffusion chambers, collected at the time of parasite recovery, from mice immunized with *Ov-*103 and formulated with each of the five adjuvants. Twenty-two cytokines were analyzed in the spleen cell supernatants and 9 were detected regardless of adjuvant used. All six of the chemokines were negative in the spleen cell supernatants. The diffusion chamber fluids were negative for all of the cytokines but had detectable levels of 5 chemokines (Table 1, S1A and S2A Tables). Based on these observations, 9 cytokines were selected for further analysis in the spleen cell supernatants and 5 chemokines were selected for analysis in the diffusion chamber fluid collected from mice immunized with *Ov-*RAL-2 or the co-administered vaccines.

In the absence of adjuvant, immunization with either *Ov-*103 or *Ov-*RAL-2 induced elevated antigen-stimulated IL-5 and IL-10 responses in the spleen cells (Table 3, S1B Table). All other cytokines measured in the spleen cell supernatants, and chemokines measured in the diffusion chamber fluid, were either not detected or responses were not different between control and immunized mice. This observation suggests that both antigens predispose towards a Th2 immune response. This was confirmed when cytokines were measured in spleen cell supernatants from mice immunized with *Ov-*103 and *Ov-*RAL-2 formulated with each of the tested adjuvants. A consistent observation was the increased production of IL-5 and IL-10, although the levels varied based on vaccine antigen and adjuvant combination. Antigen-stimulated IL-4 and IL-13 levels were elevated in most of the groups, confirming the development of Th2 responses. In addition, there was an increase in IL-6 for both antigens when formulated with all adjuvants except Advax 2. There was also an increase in IL-17A and IL-17F in mice immunized with *Ov-*103 formulated with alum or MF59 and mice immunized with RAL-2 formulated with MF59 (Table 3, S1A and S1C Table). Mice immunized with *Ov-*103/*Ov-*RAL-2 co-administered vaccines formulated with each of the three adjuvants also preferentially induced Th2 immune responses, based on the elevated levels of IL-5, IL-10 and IL-13 in the supernatants from *ex vivo* stimulated spleen cells (Table 4, S1D Table).

**Table 3:** Fold differences in mean cytokine levels from mice immunized with *Ov-*103, *Ov-*RAL-2, or co-administration of *Ov-*103 and *Ov-*RAL-2 with adjuvant.

		No Adjuvant			Ov103					OvRAL-2			
		Ov-103	Ov-RAL-2		Alum	Advax 1	Advax 2	Advax 3	MF59		Alum	Advax 2	MF59
Spleen Cell Stimulation supernatant	**IL-6**	=	=	**IL-6**	3	3	=	3	2	**IL-6**	2	=	3
	**IL-2**	=	=	**IL-2**	↓	↓	=	=	6	**IL-2**	=	=	=
	**IFN-γ**	=	=	**IFN-γ**	=	=	1.6	↓	=	**IFN-γ**	=	=	=
	**IL-4**	=	=	**IL-4**	3	=	=	=	3	**IL-4**	2	=	5
	**IL-5**	4	3	**IL-5**	27	6	2	=	9	**IL-5**	2	3	18
	**IL-10**	3	2	**IL-10**	6	4	4	3	8	**IL-10**	6	3	23
	**IL-13**	ND	ND	**IL-13**	8	2.3	=	=	3	**IL-13**	3	ND	8
	**IL-17A**	=	=	**IL-17A**	2	=	=	ND	2	**IL-17A**	=	=	3
	**IL-17F**	=	=	**IL-17F**	2	=	=	=	2	**IL-17F**	=	=	2

Fold differences, as compared to values from controls, in mean cytokine levels from stimulated spleen cell cultures from mice immunized with *Ov-*103 and *Ov-*RAL-2 with and without adjuvants, (=) same values in control and immunized mice; (↓) decrease in mean cytokine levels in immunized mice as compared to controls; (ND) not detected.

**Table 4:** Fold differences in mean cytokine levels from mice immunized with co-administration of *Ov-*103 and *Ov-*RAL-2 with adjuvant.

		Alum		Advax 2		MF59	
		Ov-103	Ov-RAL-2	Ov-103	Ov-RAL-2	Ov-103	Ov-RAL-2
Spleen Cell Stimulation supernatant	**IL-6**	3	=	2	=	3	=
	**IL-2**	2	=	2	2	=	2
	**IFN-γ**	2	=	3	2	=	=
	**IL-4**	3	6	2	2	3	3
	**IL-5**	10	15	11	=	10	9
	**IL-10**	2	3	34	3	8	10
	**IL-13**	10	5	14	ND	8	9
	**IL-17A**	=	3	3	=	3	6
	**IL-17F**	4	3	=	=	2	3

Fold differences, as compared to values from controls, in mean cytokine levels from stimulated spleen cell cultures from mice immunized with *Ov-*103 and *Ov-*RAL-2 co-administered vaccines formulated with adjuvants. (=) same values in control and immunized mice; (↓) decrease in mean cytokine levels in immunized mice as compared to controls; (ND) not detected.

Mice immunized with *Ov-*103 or *Ov-*RAL-2 without adjuvants had equivalent but low levels of the five measured chemokines in the diffusion chamber fluid, as compared to controls. Interestingly, mice immunized with *Ov-*103 formulated with each of the three adjuvants that induced protective immunity, shared the phenotype of having elevated KC and eotaxin in the parasite microenvironment, in distinction to mice immunized with *Ov-*103 and Advax 1 or CpG as adjuvants. Mice immunized with *Ov-*103 and MF59 as the adjuvant also had elevated MCP-1, MIP1α and MIP1β (Table 5, S2B Table). In comparison, mice immunized with *Ov-*RAL-2 with alum or Advax 2 as adjuvants had elevated MCP-1 and MIP1α, but did not have increased KC or eotaxin (Table 5, S2C Table). Mice immunized with the co-administered vaccine formulated with alum had elevated response to all five measured chemokines. Mice immunized with the co-administered vaccine formulated with Advax 2 had elevated levels of KC and mice immunized with MF59 as the adjuvant had elevated MCP-1 and MIP1β (Table 6, S2D Table). Correlation analyses comparing mean-parasite-recovery numbers and cytokine or chemokine levels did not reveal significant correlations.

**Table 5:** Fold differences in mean chemokine levels from mice immunized with *Ov-*103 or *Ov-*RAL-2 with and without adjuvant.

		No Adjuvant			Ov103						OvRAL-2		
		Ov-103	Ov-RAL-2		Alum	Advax 1	Advax 2	Advax 3	MF59		Alum	Advax 2	MF59
Chamber Fluid	**KC**	=	=	**KC**	7	=	3	↓	10	**KC**	=	=	=
	**MCP-1**	=	=	**MCP-1**	=	=	=	↓	9	**MCP-1**	3	2	=
	**MIP-1α**	=	=	**MIP-1α**	=	2	=	↓	3	**MIP-1α**	3	2	=
	**MIP-1β**	=	=	**MIP-1β**	=	=	=	↓	6	**MIP-1β**	6	=	=
	**Eotaxin**	=	=	**Eotaxin**	5	=	2	2	2	**Eotaxin**	=	↓	=

Fold differences, as compared to values from controls, in mean chemokine levels from diffusion chamber fluid from mice immunized with *Ov-*103 and *Ov-*RAL-2 with and without adjuvants. (=) same values in control and immunized mice; (↓) decrease in mean cytokine levels in immunized mice as compared to controls; (ND) not detected.

**Table 6:** Fold differences in mean chemokine levels from mice immunized with co-administration of *Ov-*103 and *Ov-*RAL-2 with adjuvant.

		Alum	Advax 2	MF59
**Chamber Fluid**	**KC**	2	2	=
	**MCP-1**	2	=	2
	**MIP-1α**	3	=	=
	**MIP-1β**	3	=	3
	**Eotaxin**	2	=	=

Fold differences, as compared to values from controls, in mean chemokine levels from diffusion chamber fluid from mice immunized with *Ov-*103 and *Ov-*RAL-2 co-administered vaccines formulated with adjuvants. (=) same values in control and immunized mice; (↓) decrease in mean cytokine levels in immunized mice as compared to controls; (ND) not detected.

## Discussion

Mice immunized with *Ov-*103, *Ov-*RAL-2 or with co-administered *Ov-*103/*Ov-*RAL-2 formulated with alum, Advax 2 or MF59 as the adjuvants consistently developed significant levels of both larval killing and host protection. Immunization of mice with *Ov-*103 or *Ov-*RAL-2 without adjuvant did not induce protective immunity, although immunization with the antigens stimulated recall IL-5 and IL-10 responses by spleen cells. The induction of a Th2 response by the antigens was anticipated, as evidence from both animal [27, 29] and human studies [71, 72] demonstrate that *O*. *volvulus* infection typically induces Th2-type immunity. Mice immunized with the two antigens without adjuvant did not develop antigen-specific antibody responses and there was an absence of elevated host-chemokines within the parasite microenvironment. In the absence of antibody and chemokine responses, the antigen-specific Th2 cytokine response in the spleen was insufficient to induce protective immunity to the infection.

Previous studies with *Ov-*103 and *Ov-*RAL-2 demonstrated that immunization with alum as the adjuvant induced statistically significant levels of protective immunity [33]. The goal of this study was to determine if altering the adjuvant could further enhance the induced protective immune response. Initial trials with *Ov-*103 compared five adjuvant formulations, of which three, alum, Advax 2 and MF59, induced equivalent levels of larval killing and host protection. Subsequent studies with *Ov-*RAL-2 confirmed that alum, Advax 2 and MF59 were effective adjuvants to induce equivalent levels of protective immunity. Co-administration of *Ov-*103 and *Ov-*RAL-2 with the three adjuvants induced significant larval killing and host protection, in most cases equivalent to those seen in mice receiving single antigen immunizations, as has been previously reported [31, 33]. However, in some instances there was a trend to higher levels of protective immunity in mice receiving the co-administered vaccine. The highest level of larval killing was 47% achieved in mice immunized with the two antigens with Advax 2 as the adjuvant, with some individual animals in this group achieving levels of larval killing of ~90%, which is higher than the maximal levels of larval killing achieved in any of the other single or double antigen vaccine groups. The highest level of host protection of 100% was seen in mice immunized with co-administered vaccine plus alum. Similarly, *BM-103* and *Bm*-RAL-2, injected as a fusion protein or concurrently, induced more consistent and enhanced levels of protective immunity in gerbils to *B*. *malayi*, as compared to levels achieved with individual antigens [34]. Both of the metrics used in this study, larval killing and host protection, are integral in the evaluation of a vaccine. A reduction in worm burden of approximately 50% would translate into a significant decrease in disease in the vaccinated individual and a reduction in potential transmission of infection. Host protection of 100% indicates that all vaccinated individuals responded in an efficacious manner to the vaccine and reduced infection burden, which is an important indicator of the robustness of the vaccine.

The dominant antibody isotype that was produced after immunization with *Ov-*103 and the five adjuvants and *Ov-*RAL-2 with the three adjuvants was IgG1. Only vaccines with Advax-2 induced significant IgG2a/b responses, consistent with the mixed Th1/Th2 response previously reported for this adjuvant [57–59]. The IgG1-dominated response could be predicted based on the Th2 nature of the response induced by *Ov-*103 and *Ov-*RAL-2 antigens [73]. Antigen-specific IgE was not measureable in any of the immunized mice in this study. However, IgE was shown to be a component of the protective immune response to *O*. *volvulus* induced by irradiated larvae [26]. The protective immune response induced with the recombinant antigens therefore differs from the mechanism induced by irradiated larvae. The absence of an IgE response induced by *Ov-*103 and *Ov-*RAL-2 is a significant benefit, as it reduces the possibility of adverse allergic responses when the vaccine is used clinically [74]. Finally, antigen-specific IgM levels at the terminal bleed were equivalent in control and immunized mice. This suggests that parasites within the diffusion chambers implanted in control mice released the antigens that induced an IgM response rather than this reflecting a response primed by the vaccine.

Immunization with *Ov-*RAL-2 stimulated much higher antibody endpoint titers than *Ov-*103, but this did not translate to higher levels of protective immunity. Likewise, immunization with co-administered *Ov-*103/*Ov-*RAL-2 vaccines further increased the antibody endpoint titers but with inconsistent increases in protective immunity. Correlation analysis using both pre-challenge and study termination sera was performed in an attempt to identify potential components of the killing mechanism. A clear relationship between antibody titer and protective immunity was not observed. Antibody is required for killing larval *O*. *volvulus* after immunization with irradiated larvae [26]. The quantity of antibody may not be a limiting factor to kill the larvae, with only a low titer of antibody required to effect larvae killing and hence any potential correlations were possibly obscured in the present study.

In previous studies mice were immunized with *Ov-*103 and *Ov-*RAL-2 as fusion proteins with alum as the adjuvant. The levels of larval killing (11–21%) and host protection (45–58%) [33] were significantly less than observed in the present study, where the two antigens were co-administered in separate sites. Immunization of mice with both antigens, either as a fusion protein [33] or as co-administration, resulted in significantly higher antibody endpoint titers as compared to mice immunized with the antigens individually. Apparently, the two antigens act synergistically to boost the antibody response to the reciprocal antigen. This is in distinction to other *O*. *volvulus* antigens that were found to compete with each other in vaccines resulting in reduced antibody titers [31]. Changing the route of immunization from subcutaneous, used in the previous studies [31, 33], to intramuscular, used in the present study, may have enhanced the protective immune response.

Immunization of mice with the *Ov-*103 or *Ov-*RAL-2 without adjuvant induced spleen cells to produce Th2 cytokines. A consistent observation regarding *Ov-*103 and/or *Ov-*RAL-2 in combination with the different adjuvants, was the development of Th2 immune responses based on the presence of the cytokines IL-4, IL-5, IL-10 and IL-13 in supernatants from re-stimulated spleen cells. It was predicted that the adjuvants would govern the immune response with alum stimulating a restricted Th2 response [46–50], Advax 1 stimulating mixed Th1 and Th2 responses [58, 59], Advax 2 stimulating an increased Th1 response while retaining the Th2 response, CpG stimulating a Th1 response [60, 61], and MF59 stimulating a mixed Th1/Th2 response. Apparently, the Th2 nature of the antigens and the larval challenge was sufficient to dominate the immune response even under the pressure produced by the more Th1 biased adjuvants. The cytokine recall response in mice was limited to Th2 cytokines, with the exception of mice immunized with Advax 2. Two to three fold increases in the IFNγ recall responses were seen in mice immunized with Advax 2 plus either *Ov-*103 or the co-administered vaccine. Antibody responses in mice immunized with *Ov-*103, *Ov-*RAL-2 or the co-administration with Advax 2 resulted in a combined IgG1 and IgG2a/b response, consistent with a mixed Th1/Th2 response. Immunizing mice with inulin as the adjuvant with other filarial antigens derived from *B*. *malayi*, demonstrated that the adjuvant induced a balanced Th1/Th2 response [75]. In the present study, a limited Th2 cytokine response and positive IgG1 titers was seen in mice immunized with *Ov-*103 and Advax 1 or CpG, yet parasite killing was absent. Surprisingly, the CpG adjuvant was unable, despite its normal Th1 bias, to induce an IFNγ response to *Ov-*103 or IgG2 isotype switching, consistent with *Ov-*103 antigen imparting an overwhelming Th2 bias to the adaptive immune response.

Examination of the diffusion chamber contents allowed analysis of the immune response in the parasite microenvironment. Differential cell analyses were performed and relationships were not seen between specific cell types and the presence of protective immunity in mice. In addition, differences were not seen between the numbers of cells that migrated into the diffusion chambers implanted in control and immunized mice. As an alternative approach to determine the effector cells involved with parasite killing, chemokine levels were measured in the fluid found in the diffusion chambers in which the parasites were implanted. A similar approach has been utilized in studying serum from patients with occult infections with *O*. *volvulus*. With expiring microfilariae infections, MIP-1 α and MIP-1β levels increased while after treatment with ivermectin, eotaxin and MCP-1 increased, which may have attracted effector monocytes and eosinophils to clear the microfilariae from the skin of the patients [76]. Mice immunized with *Ov-*103 formulated with alum, Advax 2 or MF59, in which there was protective immunity, had increased levels of the chemokines KC and eotaxin as compared to controls. These increases were not seen in mice immunized with *Ov-*103 alone or when formulated with Advax 1 and CpG, which suggests that either these chemokines were critical for the killing response induced by *Ov-*103 or were produced as a secondary response to larval killing. KC is involved in the activation and chemotaxis of neutrophils [77, 78]. Eotaxin is a potent chemoattractant of eosinophils and basophils by binding CCR3 [79, 80]. Based on the chemokine observations, we hypothesize that protective immunity induced by *Ov-*103 formulated with alum, Advax 2 or MF59 requires neutrophils and/or eosinophils as effector cells that collaborate with antibody. Neutrophils with antibody have been shown to be effective at killing larval *O*. *volvulus in vitro* [81] and *in vivo* studies have shown that eosinophils with antibody are capable of killing *O*. *volvulus* larvae [26].

Elevated levels of the chemokines MIP-1α and MCP-1 were found in diffusion chambers recovered from mice with protective immunity induced by *Ov-*RAL-2, but not *Ov-*103, when formulated with alum or Advax 2. This observation suggests that the mechanism of protective immunity induced by *Ov-*RAL-2 differs from the mechanism induced by *Ov-*103. MIP-1α is involved in the recruitment and activation of granulocytes including neutrophils during the acute inflammatory response [82–84]. MCP-1 exhibits a chemotactic activity for monocytes and basophils but not for neutrophils or eosinophils [80, 85, 86]. Chemokine results from protected mice vaccinated with *Ov-*RAL-2 formulated with alum or Advax 2 suggest that the effector cells required for protective immunity induced by *Ov-*RAL-2 might be macrophages and/or neutrophils. Macrophages from mice and humans have been shown to kill nematode larvae in both the innate and adaptive immune response. Furthermore, optimal killing required both neutrophils and macrophages to be active [87].

Chemokine levels in diffusion chambers from mice immunized with the co-administered vaccine displayed disparate responses. In mice immunized with the co-administered vaccine formulated with Advax 2 and MF59, chemokine levels were similar to those seen in mice immunized with *Ov-*103 but different from that seen in mice immunized with *Ov-*RAL-2, suggesting that with these adjuvants *Ov-*103 is the dominant antigen. Chemokines in mice immunized with the co-administered vaccine formulated with alum, had chemokines found to be associated with both of the individual antigen vaccines. The combined chemokine response might explain the development of 100% host protection in mice immunized with *Ov-*103 and *Ov-*RAL-2 co-administered vaccines formulated with alum.

Cytokines found in the spleen-cell supernatants also support a role for eosinophils, neutrophils and macrophages in the protective immune responses. All of the immunized mice had elevated levels of IL-5, which has been shown to be required for eosinophil differentiation, maturation and survival [88]. Mice immunized with *Ov-*103 formulated with alum or MF59 and mice immunized with *Ov-*RAL-2 formulated with MF59 also had increases in IL17A/F, consistent with a Th17 response. IL-17 has been shown to promote the production of IL-6, IL-8, G-CSF, and GM-CSF [89–91], and was demonstrated to induce numerous proinflammatory chemokines including MCP-1 and GRO-α that lead to monocyte and neutrophil recruitment [92–94]. However, the fact that mice immunized with vaccines formulated with Advax 2 induced robust larval killing despite not inducing IL17 suggests IL17 is not critical for vaccine protection. Immunization of mice with *Ov-*103 or *Ov-*RAL-2 formulated with alum or MF59 also resulted in production of IL-6 from stimulated spleen cells. IL-6 has been shown to play a crucial role in both innate and adaptive immune response, and along with IL-1β and TNFα attracts neutrophils during the initial phase of the immune response. Following the initial response, IL-6 trans-signaling leads to a switch from neutrophil recruitment to monocyte recruitment by suppressing many cytokines involved in the recruitment of neutrophils. Further, it upregulates a number of monocyte-attracting chemokines such as MCP-1 [95–98]. However, the protection in the Advax 2 group in the absence of a significant IL-6 response again argues against a key role of IL-6 in larval killing.

In conclusion, immunizing mice with the recombinant antigens *Ov-*103 and *Ov-*RAL-2 formulated with alum, Advax 2 or MF59 induced significant levels of larval killing and host protection. The immune response was biased towards Th2 with all three adjuvants, with IgG1 the dominant antibody induced in response to both antigens. Only Advax 2 induced high levels of IgG1 and IgG2a/b antibodies to both antigens. Co-administration of *Ov-*103/*Ov-*RAL-2 formulated with each of the three adjuvants induced larval killing, improved host protection and significantly increased antibody titers. Based on chemokine results, it appears that neutrophils and eosinophils may play a role in protective immunity induced by *Ov-*103 and macrophages and neutrophils in protective immunity induced by *Ov-*RAL-2. The co-administered vaccines, comprised of immunizations with both *Ov-*103 and *Ov-*RAL-2 antigens, had enhanced efficacy in controlling infections with *O*. *volvulus*, potentially based on the collaboration of two unique but synergistic killing mechanisms. Therefore, the mechanism of protective immunity induced by *Ov-*103 and *Ov-*RAL-2 formulated with alum, Advax 2 or MF59 appears to be multifactorial, with roles for antibody, cytokines, chemokines and specific effector cells. Further improving these vaccines will require strategies to optimize levels of all these protective mechanisms that contribute to larval killing.

## Supporting Information

S1 Table Cytokine levels produced by antigen-stimulated spleen cell cultures derived from mice immunized with *Ov-*103 and *Ov-*RAL-2. Data presented are means ± standard deviations. Measurement of 22 cytokine responses from mice immunized with *Ov*-103 in conjunction with one of five adjuvants (A). Measurement of 9 cytokine responses from mice immunized with *Ov*-103 or *Ov-RAL-2* without adjuvant (B). Measurement of 9 cytokine responses from mice immunized with *Ov*-RAL-2 in conjunction with one of three adjuvants (C). Measurement of 9 cytokine responses from mice immunized with co-administered *Ov*-103 and *Ov*-RAL-2 in conjunction with one of three adjuvants (D).
(PDF)

S2 Table Chemokine measurements from diffusion chamber fluids recovered from mice immunized with *Ov-*103 and *Ov-*RAL-2. Data presented are means ± standard deviations. Chemokine responses from mice immunized with *Ov*-103 in conjunction with one of five adjuvants (A). Chemokine responses from mice immunized with *Ov*-103 or *Ov-RAL-2* without adjuvant (B). Chemokine responses from mice immunized with *Ov*-RAL-2 in conjunction with one of three adjuvants (D). Chemokine responses from mice immunized with co-administered *Ov*-103 and *Ov*-RAL-2 in conjunction with one of three adjuvants (E).
(PDF)
