# Supplementary material for: The Immunomodulatory Role of Adjuvants in Vaccines Formulated with the Recombinant Antigens Ov-103 and Ov-RAL-2 against Onchocerca volvulus in Mice
Source: PLoS Negl Trop Dis. 2016 Jul 7;10(7):e0004797. doi: 10.1371/journal.pntd.0004797 (PMC4936747; doi:10.1371/journal.pntd.0004797)
Supplement: S1 Table — Data presented are means ± standard deviations. Measurement of 22 cytokine responses from mice immunized with Ov-103 in conjunction with one of five adjuvants (A). Measurement of 9 cytokine responses from mice immunized with Ov-103 or Ov-RAL-2 without adjuvant (B). Measurement of 9 cytokine responses from mice immunized with Ov-RAL-2 in conjunction with one of three adjuvants (C). Measurement of 9 cytokine responses from mice immunized with co-administered Ov-103 and Ov-RAL-2 in conjunction with one of three adjuvants (D). (PDF) [file pntd.0004797.s001.pdf]

Supplement Table 1

A.

Ov-103										
	**Alum**		**Advax 1**		**Advax 2**		**CPG**		**MF59**	
	**Control**	**Immune**	**Control**	**Immune**	**Control**	**Immune**	**Control**	**Immune**	**Control**	**Immune**
**IL-6**	1325 ± 1229	3973 ± 3124	1490 ± 865	3578 ± 1416	1372 ± 1277	1524 ± 1255	1916 ± 1973	5603 ± 1954	1401 ± 783	3337 ± 1190
**IL-2**	195 ± 103	87 ± 63	748 ± 198	308 ± 204	194 ± 166	111 ± 65	362 ± 321	194 ± 213	1473 ± 502	268 ± 132
**IFN-g**	4437 ± 2642	4128 ± 3089	6320 ± 674	4928 ± 1746	5645 ± 3081	9612 ± 3929	5712 ± 2979	3002 ± 2229	8024 ± 3302	5139 ± 1325
**IL-4**	247 ± 121	731 ± 370	728 ± 235	1061 ± 359	673 ± 439	709 ± 445	661 ± 360	886 ± 190	895 ± 252	2229 ± 710
**IL-5**	77 ± 54	2104 ± 1532	246 ± 171	1364 ± 463	266 ± 149	601 ± 266	543 ± 816	826 ± 297	1667 ± 1199	14366 ± 1762
**IL-10**	169 ± 76	1065 ± 668	465 ± 169	1610 ± 665	191 ± 139	676 ± 449	318 ± 450	887 ± 494	639 ± 317	4810 ± 1419
**IL-13**	238 ± 259	1893 ± 1411	1252 ± 509	2847 ± 1082	1084 ± 446	1859 ± 1239	1928 ± 2838	1919 ± 1629	1797 ± 1012	5673 ± 1590
**IL-17A**	23233 ± 9236	55294 ± 44652	1217 ± 923	1244 ± 939	1265 ± 1048	1602 ± 1854	ND	ND	1247 ± 1499	2675 ± 1278
**IL-17F**	66997 ± 39361	116444 ± 56252	1451 ± 666	1263 ± 670	1602 ± 990	1405 ± 1372	612 ± 853	521 ± 584	1208 ± 888	2434 ± 742
**GMCSF**	*890 ± 490*	*461 ± 368*	*1685 ± 386*	*962 ± 577*	*2173 ± 729*	*1884 ± 1147*	*1818 ± 1097*	*788 ± 718*	*2320 ± 620*	*449 ± 181*
**IL-1α**	*112 ± 38*	*149 ± 91*	*219 ± 93*	*302 ± 168*	*73 ± 20*	*80 ± 23*	*146 ± 75*	*184 ± 76*	*49 ± 8*	*45 ± 11*
**IL-3**	*5308 ± 3223*	*2947 ± 1784*	*7239 ± 1287*	*5676 ± 2007*	*5677 ± 2451*	*5052 ± 2502*	*6080 ± 3260*	*4303 ± 2456*	*11114 ± 2970*	*4738 ± 1521*
**IL-12p40**	*ND*	*ND*	*ND*	*ND*	*ND*	*ND*	*ND*	*ND*	*23 ± 16*	*17 ± 1*
**IL-12p70**	*7 ± 7*	*3 ± 1*	*ND*	*ND*	*ND*	*ND*	*ND*	*ND*	*ND*	*ND*
**TNF-α**	*170 ± 119*	*130 ± 82*	*151 ± 58*	*161 ± 44*	*72 ± 16*	*72 ± 24*	*105 ± 31*	*145 ± 53*	*134 ± 17*	*143 ± 16*
**IL-17/IL-25**	*ND*	*ND*	*ND*	*ND*	*ND*	*ND*	*ND*	*ND*	*ND*	*ND*
**IL-21**	*ND*	*ND*	*ND*	*ND*	*ND*	*ND*	*ND*	*ND*	*ND*	*ND*
**IL-22**	*258292 ± 135440*	*376860 ± 120734*	*1390 ± 278*	*1559 ± 341*	*1544 ± 309*	*1298 ± 557*	*682 ± 378*	*741 ± 264*	*1644 ± 561*	*2145 ± 163*
**IL-28b**	*486 ± 27*	*510 ± 36*	*127 ± 21*	*121 ± 15*	*ND*	*ND*	*ND*	*ND*	*ND*	*ND*
**IL-23**	*ND*	*ND*	*ND*	*ND*	*ND*	*ND*	*ND*	*ND*	*ND*	*ND*
**IL-33**	*ND*	*ND*	*ND*	*ND*	*ND*	*ND*	*ND*	*ND*	*ND*	*ND*
**TNF-β**	*ND*	*ND*	*ND*	*ND*	*ND*	*ND*	*ND*	*ND*	*740 ± 121*	*ND*

Supplement Table 1 continued

B.

	No Adjuvant		
	Control	Ov-103	Ov-RAL-2
IL-6	919 ± 701	699 ± 652	1428 ± 850
IL-2	115 ± 67	165 ± 35	102 ± 55
IFN-g	2686 ± 727	2750 ± 949	2403 ± 533
IL-4	263 ± 252	448 ± 147	267 ± 87
IL-5	168 ± 244	679 ± 157	444 ± 262
IL-10	ND	ND	ND
IL-13	ND	ND	ND
IL-17A	1907 ± 3414	101 ± 55	619 ± 423
IL-17F	822 ± 740	192 ± 123	649 ± 298

C.

	Ov-RAL-2					
	Alum		Advax 2		MF59	
	Control	Immune	Control	Immune	Control	Immune
IL-6	1019 ± 340	2203 ± 797	1184 ± 491	2516 ± 936	1093 ± 704	2973 ± 2231
IL-2	133 ± 86	159 ± 116	85 ± 46	43 ± 41	173 ± 104	111 ± 71
IFN-g	3291 ± 2382	1950 ± 758	2087 ± 747	3349 ± 1658	2734 ± 1149	2081 ± 777
IL-4	231 ± 183	460 ± 200	86 ± 55	76 ± 51	268 ± 308	1214 ± 160
IL-5	231 ± 438	553 ± 299	19 ± 13	49 ± 48	354 ± 718	6530 ± 1450
IL-10	34 ± 42	196 ± 185	13 ± 7	40 ± 38	84 ± 119	1886 ± 1308
IL-13	297 ± 406	735 ± 327	ND	ND	ND	3105 ± 3324
IL-17A	1636 ± 740	1928 ± 1488	1773 ± 1227	1534 ± 1081	871 ± 570	2765 ± 1444
IL-17F	786 ± 225	1056 ± 444	818 ± 442	510 ± 440	622 ± 433	1490 ± 391

D.

	Ov-103/Ov-RAL-2											
	Alum				Advax 2				Mf59			
	Ov-103		Ov-RAL-2		Ov-103		Ov-RAL-2		Ov-103		Ov-RAL-2	
	Control	Immune	Control	Immune	Control	Immune	Control	Immune	Control	Immune	Control	Immune
IL-6	1085 ± 1172	2997 ± 2470	8379 ± 4181	9693 ± 2297	1006 ± 487	2196 ± 1966	3017 ± 2902	3809 ± 816	378 ± 361	1233 ± 1264	3680 ± 3235	5308 ± 2930
IL-2	349 ± 423	518 ± 326	312 ± 603	313 ± 329	338 ± 156	616 ± 677	100 ± 53	225 ± 187	169 ± 82	227 ± 175	114 ± 28	201 ± 165
IFN-g	1320 ± 1070	2111 ± 955	2868 ± 1680	3289 ± 1073	2248 ± 2334	5911 ± 1382	2729 ± 1438	4706 ± 1953	1214 ± 970	1664 ± 1045	1737 ± 700	2412 ± 921
IL-4	391 ± 376	1168 ± 546	227 ± 162	1338 ± 580	432 ± 275	866 ± 618	271 ± 447	455 ± 416	206 ± 174	657 ± 238	274 ± 158	860 ± 364
IL-5	40 ± 94	397 ± 234	20 ± 46	289 ± 179	14 ± 14	154 ± 192	92 ± 224	71 ± 149	40 ± 103	380 ± 181	44 ± 103	391 ± 196
IL-10	132 ± 190	307 ± 270	109 ± 177	318 ± 164	ND	341 ± 333	54 ± 76	135 ± 90	ND	340 ± 194	64 ± 51	645 ± 393
IL-13	ND	300 ± 229	ND	163 ± 135	ND	416 ± 598	135 ± 90	ND	ND	228 ± 147	ND	255 ± 127
IL-17A	1140 ±2673	1344 ± 1452	3069 ± 5720	7315 ± 6432	1255 ± 1356	3297 ± 3183	1025 ± 820	1235 ± 861	154 ± 117	521 ± 408	1032 ± 846	6423 ± 7543
IL-17F	441 ± 671	441 ± 671	2507 ± 2854	6752 ± 1809	1159 ± 1036	1674 ± 1006	933 ± 661	931 ± 589	232 ± 187	551 ± 332	1442 ± 1201	3826 ± 3845
