# Supplementary material for: The Immunomodulatory Role of Adjuvants in Vaccines Formulated with the Recombinant Antigens Ov-103 and Ov-RAL-2 against Onchocerca volvulus in Mice
Source: PLoS Negl Trop Dis. 2016 Jul 7;10(7):e0004797. doi: 10.1371/journal.pntd.0004797 (PMC4936747; doi:10.1371/journal.pntd.0004797)
Supplement: S2 Table — Data presented are means ± standard deviations. Chemokine responses from mice immunized with Ov-103 in conjunction with one of five adjuvants (A). Chemokine responses from mice immunized with Ov-103 or Ov-RAL-2 without adjuvant (B). Chemokine responses from mice immunized with Ov-RAL-2 in conjunction with one of three adjuvants (D). Chemokine responses from mice immunized with co-administered Ov-103 and Ov-RAL-2 in conjunction with one of three adjuvants (E). (PDF) [file pntd.0004797.s002.pdf]

Supplement Table 2

A.

	Ov103									
	**Alum**		**Advax 1**		**Advax 2**		**CPG**		**MF59**	
	**Control**	**Immune**	**Control**	**Immune**	**Control**	**Immune**	**Control**	**Immune**	**Control**	**Immune**
**KC**	69 ± 29	473 ± 948	184 ± 147	129 ± 132	45 ± 29	130 ± 160	394 ± 702	104 ± 117	109 ± 116	1056 ± 1381
**MCP-1**	1032 ± 1879	853 ± 1457	1057 ± 1638	1447 ± 1402	890 ± 923	730 ± 853	449 ± 408	191 ± 305	214 ± 226	2018 ± 3509
**MIP-1α**	247 ± 287	192 ± 422	223 ± 275	439 ± 329	348 ± 373	250 ± 295	321 ± 371	117 ± 118	399 ± 625	1121 ± 1766
**MIP-1β**	173 ± 175	255 ± 564	175 ± 249	275 ± 198	250 ± 267	221 ± 272	240 ± 343	62 ± 51	225 ± 356	1432 ± 1968
**Eotaxin**	60 ± 32	309 ± 402	130 ± 87	153 ± 246	36 ± 25	73 ± 70	56 ± 40	123 ± 221	44 ± 25	94 ± 123
**Rantes**	ND	ND			ND	ND			ND	ND

B.

	No Adjuvant		
	**Control**	**Ov-103**	**Ov-RAL-2**
**KC**	1537 ± 2723	480 ± 403	460 ± 329
**MCP-1**	697 ± 505	1704 ± 3079	833 ± 361
**MIP-1α**	990 ± 1778	809 ± 1203	500 ± 279
**MIP-1β**	131 ± 94	434 ± 610	299 ± 132
**Eotaxin**	252 ± 100	232 ± 71	388 ± 278

C.

	OvRAL-2					
	**Alum**		**Advax 2**		**MF59**	
	**Control**	**Immune**	**Control**	**Immune**	**Control**	**Immune**
**KC**	708 ± 624	1074 ± 1584	598 ± 1011	747 ± 662	397 ± 388	540 ± 768
**MCP-1**	433 ± 220	1430 ± 1929	357 ± 140	710 ± 1078	872 ± 820	643 ± 429
**MIP-1α**	295 ± 170	759 ± 966	278 ± 121	600 ± 475	440 ± 319	400 ± 187
**MIP-1β**	185 ± 76	1097 ± 2662	238 ± 90	338 ± 219	318 ± 222	301 ± 164
**Eotaxin**	286 ± 270	239 ± 130	386 ± 184	139 ± 102	149 ± 125	149 ± 69

D.

	Ov-103/Ov-RAL-2					
	**Alum**		**Advax 2**		**MF59**	
	**Control**	**Immune**	**Control**	**Immune**	**Control**	**Immune**
**KC**	468 ± 530	866 ± 1341	535 ± 548	1148 ± 1068	1082 ± 712	1247 ± 1765
**MCP-1**	820 ± 812	1209 ± 942	708 ± 679	827 ± 635	1114 ± 648	2546 ± 3446
**MIP-1α**	269 ± 194	719 ± 544	221 ± 120	284 ± 177	383 ± 312	339 ± 416
**MIP-1β**	213 ± 108	559 ± 477	246 ± 121	279 ± 143	308 ± 163	796 ± 938
**Eotaxin**	127 ± 106	215 ± 430	213 ± 180	220 ± 162	352 ± 302	282 ± 142
